# Development of Deep Learning with RDA U-Net Network for Bladder Cancer Segmentation

**DOI:** 10.3390/cancers15041343

**Published:** 2023-02-20

**Authors:** Ming-Chan Lee, Shao-Yu Wang, Cheng-Tang Pan, Ming-Yi Chien, Wei-Ming Li, Jin-Hao Xu, Chi-Hung Luo, Yow-Ling Shiue

**Affiliations:** 1 Department of Mechanical and Electro-Mechanical Engineering, National Sun Yat-sen University, Kaohsiung 804, Taiwan; 2 Department of Mechanical Engineering, National United University, Miaoli 360, Taiwan; 3 Institute of Advanced Semiconductor Packaging and Testing, College of Semiconductor and Advanced Technology Research, National Sun Yat-sen University, Kaohsiung 804, Taiwan; 4 Department of Urology, Kaohsiung Medical University, Kaohsiung 807, Taiwan; 5 Department of Medicine Chest, Kaohsiung Armed Forces General Hospital, Kaohsiung 802, Taiwan; 6 Institute of Biomedical Sciences, National Sun Yat-sen University, Kaohsiung 804, Taiwan; 7 Institute of Precision Medicine, National Sun Yat-sen University, Kaohsiung 804, Taiwan

**Keywords:** computed tomography, U-Net, bladder cancer, ResNet, DenseNet

## Abstract

**Simple Summary:**

This study proposed the “Residual-Dense-Attention” (RDA) U-Net model architecture to automatically segment organs and lesions in computed tomography (CT) images. The RDA U-Net used ResBlock and DenseBlock at the encoder. Attention gates were used at the decoder position to help the model suppress irrelevant areas of the CT image. Forty-one patients’ bladder images were provided for training. The RDA U-Net provided faster but satisfactory segmentation results for bladder cancers and lesions.

**Abstract:**

In today’s high-order health examination, imaging examination accounts for a large proportion. Computed tomography (CT), which can detect the whole body, uses X-rays to penetrate the human body to obtain images. Its presentation is a high-resolution black-and-white image composed of gray scales. It is expected to assist doctors in making judgments through deep learning based on the image recognition technology of artificial intelligence. It used CT images to identify the bladder and lesions and then segmented them in the images. The images can achieve high accuracy without using a developer. In this study, the U-Net neural network, commonly used in the medical field, was used to extend the encoder position in combination with the ResBlock in ResNet and the Dense Block in DenseNet, so that the training could maintain the training parameters while reducing the overall identification operation time. The decoder could be used in combination with Attention Gates to suppress the irrelevant areas of the image while paying attention to significant features. Combined with the above algorithm, we proposed a Residual-Dense Attention (RDA) U-Net model, which was used to identify organs and lesions from CT images of abdominal scans. The accuracy (*ACC*) of using this model for the bladder and its lesions was 96% and 93%, respectively. The values of Intersection over Union (*IoU*) were 0.9505 and 0.8024, respectively. Average Hausdorff distance (*AVGDIST*) was as low as 0.02 and 0.12, respectively, and the overall training time was reduced by up to 44% compared with other convolution neural networks.

## 1. Introduction

With the improvement of living standards and lifestyle changes, modern people have unhealthy lifestyles, such as smoking, excessive drinking, staying up late, or having an unbalanced diet, etc., which lead to a high incidence of cancer and cardiovascular diseases. The bladder is composed of a muscular cystic structure and is an organ prone to cancer. A layer of urothelial cell tissue on the surface contacts the interior. If this cell tissue develops cancer, it is called bladder cancer [1]. Among them, the superficial type that is easy to cure accounts for 75–85% [2], and the malignant type that is highly dangerous and easy to invade or metastasize accounts for 15–25% [2], and it is difficult to be discovered in the early stage. Because the bladder size of each patient is very different, it is difficult to stage bladder cancer [3]. The staging will be based on the extent to which the cancer cells have invaded the bladder wall and muscle layer, or whether they have invaded the lymph or organ metastasis. From stage 0 to stage 4, stage 0 can be called “superficial bladder cancer”, which means that the cancer cells only exist in the mucosal layer of the bladder but not deep into the muscle layer; stage 1 is when the cancer cells invade the mucosal layer, the muscle layer, and lymph nodes have not yet been invaded; the second stage is that the cancer cells have invaded the muscle layer but not invaded the fat layer yet; the third stage is that the cancer cells have invaded the muscle layer and have penetrated the surrounding fat layer, and can spread to nearby organs and lymph nodes; and in the fourth stage, the cancer cells penetrate all layers of the bladder wall and spread to the abdominal cavity and lymph nodes, among which the cancer cells have invaded the muscle layer or have metastasized (such as the lung, bone, or liver) [4].

For cancer with the highest mortality rate, the symptoms are generally not apparent at the early stage, and it is not easy to detect and treat it early. Doctors need to manually review the image results of magnetic resonance imaging (MRI) [5] and computed tomography (CT) imaging examinations to make judgments. Most tumors need further investigation. Tumor tissue biopsy [6] is the most accurate way to confirm the diagnosis, but tumor biopsy is an invasive examination with inevitable complications such as bleeding and tumor metastasis (needle tract tumor seeding). The chest and the abdominal cavity can be checked separately during the health examination. The abdominal cavity has many organs, and it is impossible to use abdominal ultrasound alone to check whether there is cancer. Therefore, it is necessary to use computerized tomography, blood tests, and biopsy. Continuous images of slices in different directions, so CT images can be used to inspect the abdominal organs of the patient’s abdomen. Therefore, if CT images can be combined with artificial intelligence deep learning for bladder identification training, doctors can achieve good results in the treatment and prevention of two types of cancer during follow-up health checkups and, at the same time, reduce the burden of manual image identification. Error rates and cases where imaging cannot be manually identified, and tissue sections are required for diagnosis occur.

CT can be used in the whole body [7]. Compared with MRI, CT is more accurate in interpreting the arterial, venous, or delayed phases. It is generally used with a contrast agent for CT images of tumors that cannot be seen with the naked eye. The contrast agent is produced by two different formulas: iodine or barium sulfate. Using both can effectively limit X-ray penetration and be used to detect abnormalities. Absorbing the contrast agent thus blocks the X-ray penetration reaction, which is different from normal cells, and thus creates a contrast [8]. After intravenous injection of the iodine-containing contrast agent, current results show that the kidneys will metabolize about 90% of the injected contrast agent, but before use, a blood test for kidney function is still required, and the use of a contrast agent may also cause side effects, ranging from diarrhea, nausea, or vomiting to dyspnea, convulsions, or cardiac arrest.

Artificial intelligence (AI) [9] aims to make computers have the same complete cognitive abilities as humans. Deep learning (DL) [10] is expected to simulate the neural network operation mode formed by neurons in the human brain [11,12]. To simulate the network composed of a single neuron, it is divided into multiple layers for simulation, usually at least one input layer, a very large multi-layer hidden layer, and one output layer. Currently, the review of clinical medical imaging data mainly relies on the analysis and observation of doctors with their own eyes. This will also affect the accuracy of the diagnosis due to the doctor’s own reading experience and subjective differences. With the increase in population aging, the number of cancer patients has not been significantly reduced, and modern medical progress has also increased the number of people tested. The amount of imaging data has increased dramatically, thereby increasing the workload of physicians, examiners, and radiologists. When deep learning is used in visual processing, image preprocessing and image labeling (ground truth) are required in the early stage. When deep learning is applied to medical image identification [12,13,14,15,16,17], the identification of CT images and their lesions is complex. The annotations need to be marked and confirmed by professional doctors to ensure the benchmark of model training.

Obayya et al. [18] established an arithmetic optimization algorithm with a deep-learning-based histopathological breast cancer classification (AOADL-HBCC) technique for healthcare decision making with a maximum accuracy of 96.77%. Zhao et al. [19] designed a COVID Net CT-236 network for COVID-19 detection based on CT images, with an accuracy of 99.2%. Zováthi et al. [20] proposed a novel approach to segment tumor and normal regions in human breast tissues by CNN, and accuracy reached 99.21%. Mortazavi-Zadeh et al. [21] used U-Net++ to segment brain tumors with an accuracy of 90.37%. It can be observed that the application of deep learning to cancer detection is becoming more common in modern medicine. Baressi Šegota et al. [22] presented a transfer learning approach to the semantic segmentation of bladder cancer masses from CT images. It achieved excellent performance with an AUCmicro of 0.9999 and σ (AUCmicro) of 0.0006. Hu et al. [23] designed a multi-scale convolution Unet (MC-Unet) for bladder cancer cell segmentation in phase contrast microscopy images. The experiment was conducted on the bladder cancer dataset to verify the performance of MC-Unet, and the results showed MC-Unet had better performance than the standard Unet. Yu et al. [24] proposed a Cascade Path Augmentation Unet (CPA-Unet) network to segment multi-regional of the bladder. CPA Unet achieved excellent segmentation results in terms of Dice similarity coefficient (*DSC*) and Hausdorff distance (HD) (inner walls: *DSC* = 98.19%, HD = 2.07 mm; outer walls: *DSC* = 82.24%, HD = 2.62 mm).

The CT images can be detected in the whole body for deep learning training [25], and the images can achieve high accuracy without using contrast agents. The bladder in the abdominal organs is used for cancer lesion inspection. The bladder usage data sets are derived from open-source websites (The Cancer Genome Atlas, TCGA) [26] and Kaohsiung Medical University. The more sources of data sets for deep learning, resulting in an increase in the number of images, compared with the results of a single type of training for the test that can be applied after training, the higher accuracy can be achieved when testing. In the field of medical image segmentation, the U-Net neural network proposed by Olaf et al. [27] is used to extend the input end, combine ResBlock [28] with Dense Block [29], and use Attention Gates at the output end. Combined with the above algorithm, a Residual-Dense-Attention (RDA) U-Net model is used as the training basis. After the bladder is trained from CT images using a convolutional neural network (CNN), lesion segmentation training is performed on the organ to provide physicians with auxiliary discrimination in a pre-diagnosed manner.

The RDA U-Net model proposed for this study used the RB and DB in the encoder part to reduce the overall training time and maintain the training parameter. At the decoder part, we combined Attention Gates, which made our model suppress the irrelevant part. Our RDA U-Net model ultimately segmented the bladder and lesions from CT images without using a contrast agent. The training time was reduced by 40% compared with other U-Net models. With the training time reduced, this model can still maintain good *ACC*.

- Improve the speed and accuracy of identifying organs and lesions without contrast agent injection.
- Compared with Attention U-Net and Attention Dense-U-Net, RDA U-Net convolution reduces the calculation time by about 40%.
- With a mixture of hospital and open-source image data, RDA U-Net can identify various abdominal organs accurately.

## 2. Method

This section will introduce the data set-up and image pre-processing, data augmentation, RDA U-Net model structure, and training data. Through these introductions, we can understand how the model works internally and how the data are processed.

### 2.1. Data Set-Up and Image Pre-Processing

**Figure 1:**
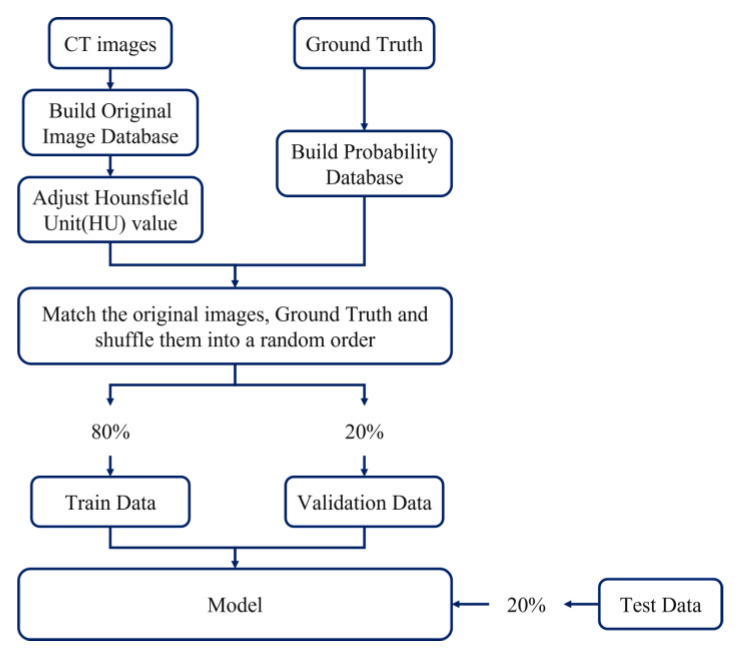
Dataset allocation and setup.

**Figure 2:**
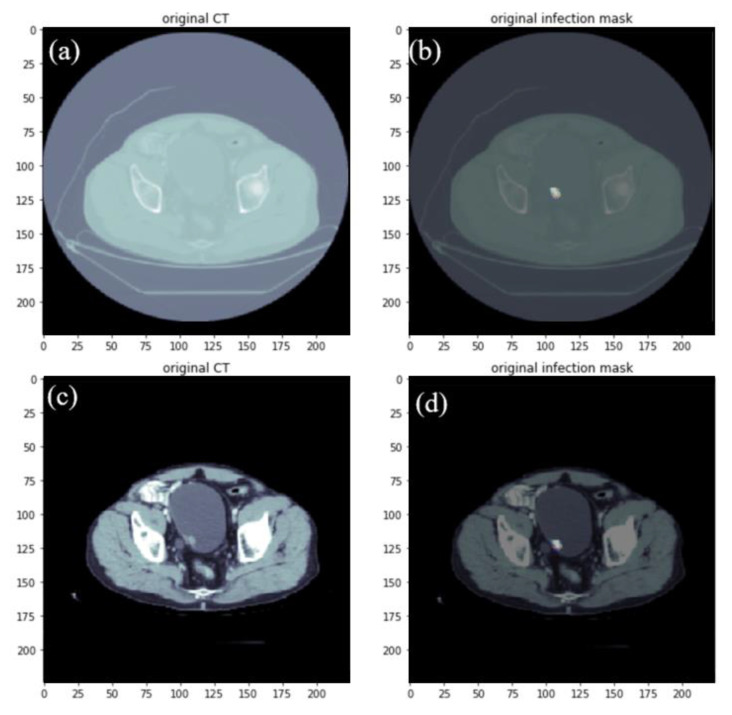
Before adjusting the HU value of image data, (**a**) the original CT of the hospital (**b**) mark the focus (white color light is the location of the focus). After adjusting the HU value of the image data (**c**), the original CT of the hospital (**d**) labeled the lesion (white color light is the location of the lesion).

The bladder dataset used in this study was from the open-source website (TCGA) [26] and hospital data (Kaohsiung Medical University). For data creation, each CT image and Ground Truth was confirmed first. The CT image with an adjusted Hounsfield unit (HU) [30] was superimposed and compared with the Ground Truth to ensure that the image and the marker were the same data, and then the compared data were randomly mixed and distributed. Through random mixing, this could slow down the occurrence of underfitting, and the data were divided into a training set and a validation set at a ratio of 8:2. Except for the validation set, it was also necessary to use images that the model had not learned, that is, images that were entirely different from the training data, as a test set to evaluate the model. The distribution and settings of the data set are shown in Figure 1. Open-source data from the Internet can only be used after screening and disclosure. The current open-source data can be roughly divided into two types. One is clear images without shrinking edges and apparent lesions, and the other is ordinary people without diseases, the way of training with these two types accounts for the vast majority of the current. Since the lesions on medical images are difficult to identify without a contrast agent, the pre-processing of the image development is used to make the image clear and adjust the Hounsfield scale, which contains the information of the HU [31]. The before and after adjustments are shown in Figure 2.

This section showed this experiment’s pre-processing, data set-up, and experimental process. A training group and validation group were obtained. We could make the focus in the picture clearer without the contrast agent.

### 2.2. Data Augmentation

Deep learning requires a large amount of data as training data. If too little data were used, it would lead to problems such as low accuracy and overfitting. However, it was not easy to obtain medical images. Therefore, it was expected to increase the total number of bladder training sheets through data augmentation; this study used the following five data augmentation methods to increase the number of training sheets:

- Size scaling: For zooming in and out of the image, this research uses the original size of the image and zooms to 120% of the original size for training.
- Rotation: The image is randomly translated and rotated at the specified angle. This research uses a rotation translation of plus or minus 15 degrees.
- Shifting: The pixel moves the image horizontally and vertically. This research moves the image on the x and y axes by plus or minus 10.
- Shearing: The random amount of the image is clipped according to the set angle range. The set value in this research is between plus and minus 5 degrees.
- Horizontal flip: The image is flipped based on the horizontal direction. In this paper, 50% of the images are converted.

**Figure 3:**
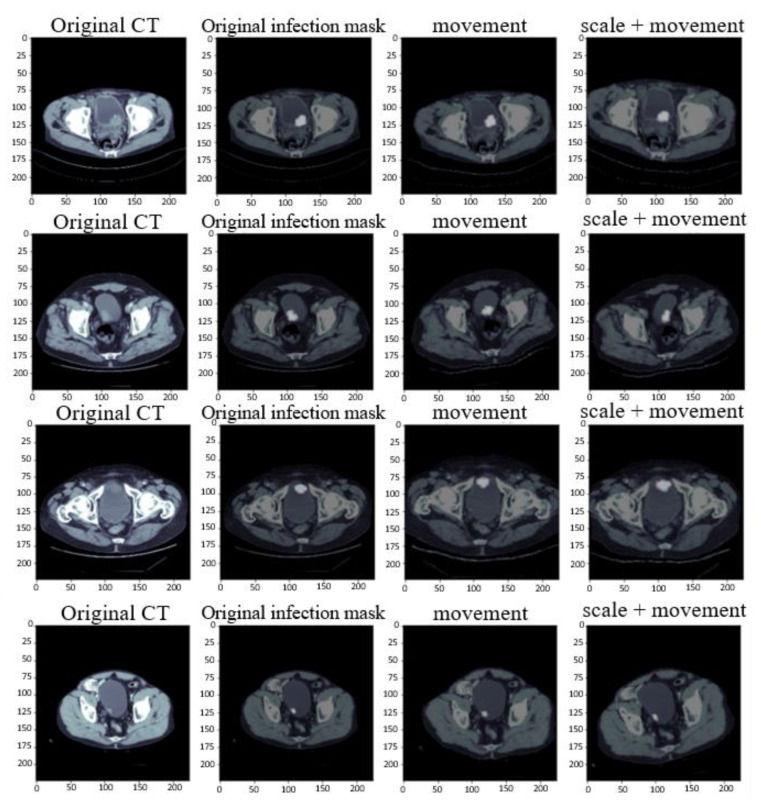
Data augmentation image of bladder lesions.

Using the above five data augmentation methods, the total number of bladder sheets was increased from 712 to 2136. In addition to adding the original image, data augmentation must also be carried out with the painted mask, and the above five methods were randomly mixed and matched. Randomly mixing and matching data with enlarged size and rotated or displaced after horizontal overturning could be generated. Because each person’s bladder had many sizes and positions, the data enhanced by random mixing would be more and more complete. The mixing and matching described above are shown in Figure 3.

### 2.3. Model Structure

The RDA U-Net model was divided into two sides: encoder and decoder. The left half of the model architecture was the encoder, which had undergone five image reductions. Respectively, two ResBlocks (RB) were collocated with one pooling [28], and three Dense Blocks (DB) were collocated with one convolution and pooling. The features in the image were captured by the convolution layer, and then the image was downsampled and reduced in size by the pooling layer. In order to preserve the image features, the image passing through the RB and DB modules was connected to the decoder feature map with the same image size and level by Skip Connection (blue dotted line arrow). The AG [32] was used at the decoder position to suppress the irrelevant areas in the image, and the useful salient features were highlighted. The recovered feature map could be retained by connecting the encoder and decoder.

**Figure 4:**
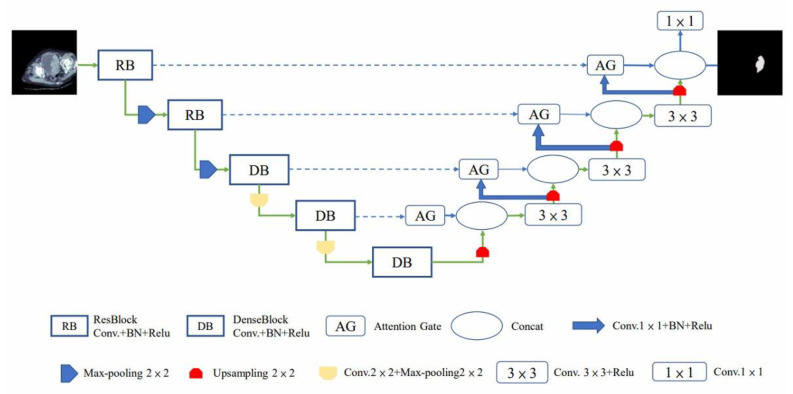
RDA U-Net model.

As shown in Figure 4, the decoder that underwent the RB and DB could reduce the image size and preserve the feature map; it contained more low-dimensional features and suppressed irrelevant regions.

### 2.4. Encoder

**Figure 5:**
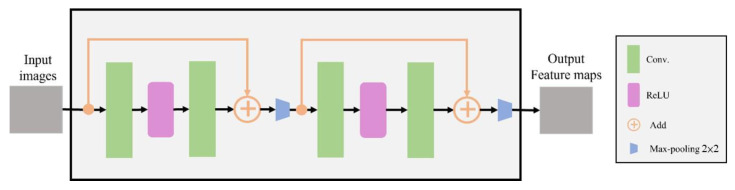
Internal structure diagram of ResBlock module.

The RB module in our convolution [33] was split in detail, as shown in Figure 5. The RB module used a shortcut to connect the input and output through “Add” in the figure. After the exact mapping across the intermediate layers, no additional parameters would be generated, and the computational complexity would not increase. The neural network would be relieved by skipping but retaining features, and the overall computing time would be reduced.

**Figure 6:**
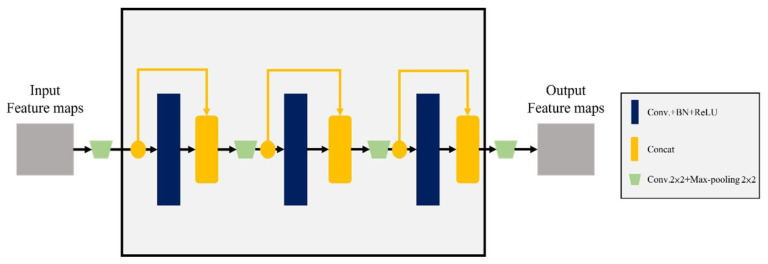
Internal structure diagram of DesBlock module.

The ResBlock module alone could reduce the overall training time. Still, because its jumping connection mode also reduced the learning ability of the network, the DB module was used as a follow-up configuration to enhance training. As shown in Figure 6, the DB module could reuse features. In the figure, all layers were connected by the concat forward propagation method, and the use of parameters was reduced by merging dimensions. The gradient of feature reduction disappears; however, this model was complicated due to the superposition of dimensions and required sufficient equipment and a long training time, so that it could be used together with the RB module.

### 2.5. Decoder

**Figure 7:**
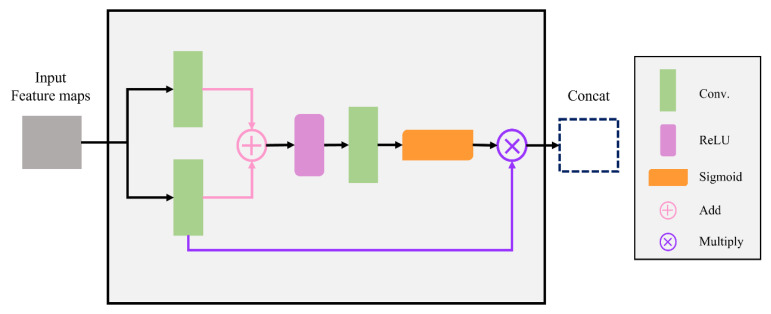
Internal structure diagram of Attention Gates module.

In addition to using the above two modules at the encoder to reduce training time and reuse features, the AG structure is shown in Figure 7. Since this module would receive the feature map of the encoder position, it was necessary first to use a convolution system to integrate the sample size, then add the image and enhance the display of the region of interest by adding the image. Finally, the excitation function operation was used to alleviate the occurrence of overfitting. Adding AG modules could improve the prediction performance under different data and training conditions.

The decoder added an AG module to enhance the display of the feature area through the “Add” in the module and then solved the occurrence of over-fitting through the Relu function.

### 2.6. Training Data

The bladder used the open-source TCGA dataset [26] on the Internet. There were 120 CT images of bladder cancer patients in the data. We excluded the images of patients with blurred images, blurred bladder muscle layers, and too-small lesions that could not be identified. Finally, 31 patients were selected for identification. And 38 patients provided by Kaohsiung Medical University (KMU) were chosen from the first to fourth phases of the recommendations given by doctors. Finally, 10 patients’ CT images were selected. A total of 41 patients were combined into a data set. Because of the small number of bladder images, data enhancement was used to increase the training data and improve the overall training results. The training data was divided into training and verification sets at an 8:2 ratio. To adapt to the model and hardware, pixel values were changed to reduce the pixel quality from 512 × 512 to 224 × 224 to improve computing efficiency.

After obtaining medical images, cases with blurred images or too small lesions were filtered out. The selected cases were divided into training sets and verification sets at a ratio of 8:2. Finally, the image quality was reduced to improve computational efficiency.

## 3. Evaluation Metrics

In this section, we will introduce the evaluation indicators. This research uses the following four indicators: Accuracy (*ACC*), Dice Score Coefficient (*DSC*), Intersection over Union (*IOU*) [34,35], and Average Hausdorff Distance (*AVGDIST*) [36,37]. Through these indicators, we can know how well our model is performing.

The following indicators could be used to evaluate the model and other basic models. We used *ACC*, *DSC*, *IoU*, and *AVGDIST* as evaluation methods. The above indicator could be calculated using the following four parameters: True positive (TP), true negative (TN), false positive (FP), and false negative (FN). The image of medical image segmentation had only two colors. One was the white image of the expected, predicted part of the lesion or organ, and the remaining image that was not this part would be the black of the background. Because of the accuracy calculation, TN had to add the correct black part to the calculation. While the black part in Ground Truth accounted for the majority, the accuracy result would be better than the value obtained by other calculation methods. Values ranged from 0 to 1, the best result was 1, and the worst was 0. If it was 1, it meant that the prediction was completely consistent with the Ground Truth. The calculation formula of *ACC* is:

(1) $$ACC=\frac{TP+TN}{TN+FP+TP+FN}$$

*DSC* was often used to calculate the segmented image standard, which was the similarity used for the two samples. The value range was 0~1. The best result of segmentation was 1, and the worst difference was 0, as shown below:

(2) $$DSC=\frac{2TP}{FP+2TP+FN}$$

*AVGDIST* divided the prediction results into black parts as 0 and white parts as 1. After the prediction results and Ground Truth were overlapped, the overlapped images were obtained, and their values were calculated. For the medical images with many black areas, the *IoU* formula for the training area was developed as follows:

(3) $$IOU=\frac{TP}{FP+TP+FN}$$

Hausdorff distance was sensitive to the segmented boundary range. This calculation would compare the boundary range distance between the training result and the actual result image. Therefore, this calculation formula was mainly used for image segmentation. The formula is as follows:

(4) $$\mathrm{Average}\;\mathrm{Hausdorff}\;\mathrm{distance}=(\frac{GtoS}{G}+\frac{GtoS}{S})/2$$

## 4. Result and Discussion

### 4.1. Parameter Setting of Experimental Learning Rate

The parameters of the bladder part convolution network used in this study were set to an epoch and batch of 200 and 8, the learning rate was 10^−3^, the epoch and batch of the lesion part were 100 and 8, and the learning rate was set to 10^−3^. The ADAM optimizer was used to update the parameters of the entire network. While 80% of the entire data set was randomly selected as the training set, the remaining 20% was used as the verification set. In addition to the validation set, it was necessary to use images that the model had not learned; that is, images completely different from the training data must be used as the test set to evaluate the model.

### 4.2. Comparison of Bladder Training Time and Convolution Parameters

In the Attention Dense-U-Net [38] proposed by Li et al. in 2019, the network structure of this collocation was more complex, so it took more time to update the network parameters in the training process. This paper chose to increase the appropriate time cost to improve the tumor’s segmentation accuracy. Compared with the convolutional network of Attention U-Net [32] and Attention Dense U-Net [38], the RDA U-Net constructed by us could achieve the accuracy of this network with less time cost, and our model convolution parameters were also higher than Attention Res U-Net [39]. The overall network training time could be reduced by the hop connection of ResBlock, and DenseBlock could maintain the convolution parameters of the overall model. Therefore, the accuracy rate was reached, and the overall training time was 44% lower than Attention Dense-U-Net, as shown in Table 1 below.

The RDA U-Net model constructed in this experiment was compared with other convolutional network models. The accuracy was similar to other models, but the training time was much reduced.

### 4.3. Residual-Dense Attention U-Net Model Convergence Curve

**Figure 8:**
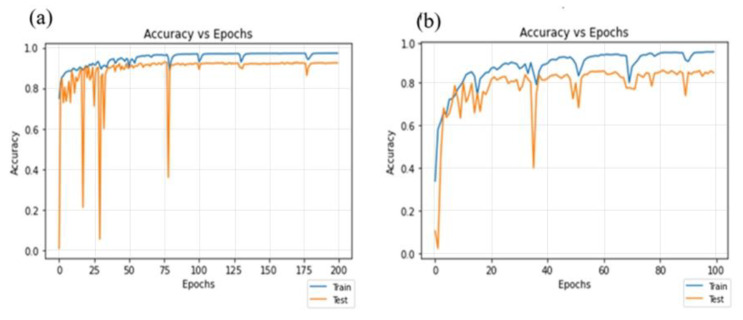
Training accuracy results of 41 patients with bladder mixed with TCGA and KMU. (**a**) Bladder; (**b**) lesion.

The data of 41 patients with TCGA and KMU were mixed, the total number of images was about 712, and the number of images was enhanced to 2136 for training with data augmentation and image pre-processing. The pixel value was changed from 512 × 512 to 224 × 224. After imaging and limiting the HU value using RDA U-Net and adjusting convolution parameters and experimental parameters, 200 epochs were used in the bladder part and 100 epochs were used in the bladder lesion part for training. The accuracy is shown in Figure 8a,b, respectively. The bladder *ACC* value was 0.9656, and the lesion *ACC* value was 0.9394.

### 4.4. Bladder and Lesion Segmentation Results

This chapter will use the above evaluation metrics to compare our convolution RDA U-Net and the following three related convolutions, namely, Attention U-Net [32], Attention Dense U-Net [38], and Attention Res U-Net [39], for segmentation capability comparison.

For the results of bladder segmentation in Table 2 and bladder lesion segmentation in Table 3, Attention U-Net had the best overall performance. And the overall error of Attention U-Net and Attention Dense U-Net in *DSC* and *IoU* was less than 2% compared with our convolution. RDA U-Net convolution was the second-best training network for the bladder. Compared with the training times of Attention U-Net and Attention Dense U-Net, RDA U-Net required less training time to achieve excellent training results.

### 4.5. Analysis of the Results of Bladder Cancer

**Figure 9:**
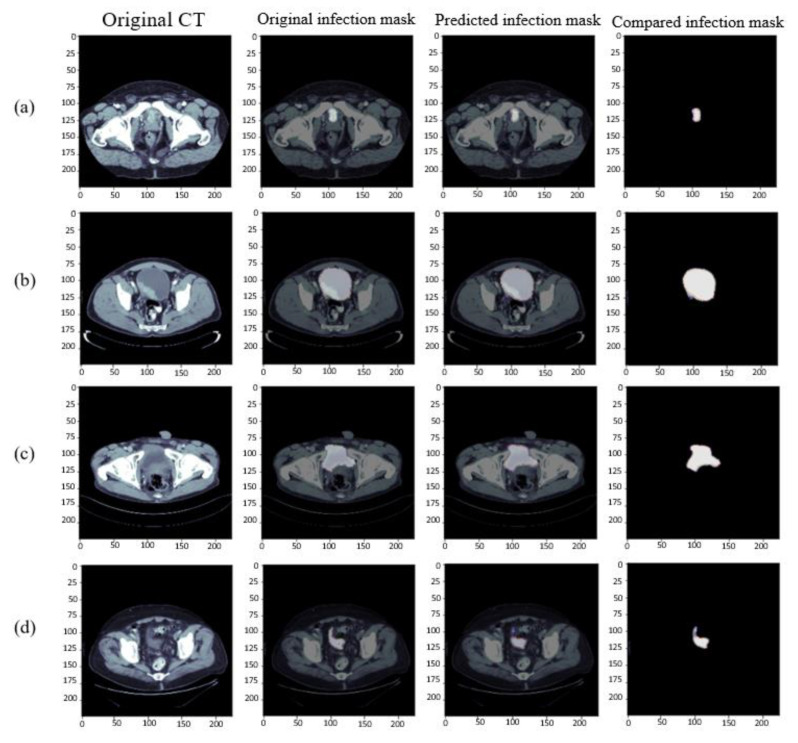
Results of bladder segmentation. From left to right, original CT images, Ground Truth, prediction results, and the superposition comparison of Ground Truth and prediction results. (**a**) Bladder near pubic junction (**b**) round bladder, (**c**) irregular bladder, and (**d**) bladder near abdomen.

The organs of the bladder would expand because they could store water, so the organs of the bladder had a considerable number of shapes, and their lesions had various conditions. The segmentation results of the bladder organs are shown in Figure 9. In the training results, it could be observed that because the last part of the bladder was close to the pubis, some patients in Figure 9a still had a bladder at this position and would be blocked by the pubis. However, it could be observed from the figure that our model also had a certain learning ability in this part. The bladder in Figure 9b,c, in addition to the round bladder, had the above irregular shape bladder. It can be seen from the figure that the RDA U-Net model also has a good segmentation effect on the bladder of various shapes. In addition to being close to the pubis, the other end of the bladder was close to the position of the abdominal intestine. Thus, the top fuzzy bladder near the abdomen in Figure 9d was generated. Although the central bladder position was learned, there were still omissions. The segmentation of bladder position could increase the training image of pubic and abdominal positions to facilitate better learning of the convolution network.

The shape of the bladder would change all the time. It could be seen from the figure that there were several forms; the lesions also had a variety of forms. However, RDA U-NET also had a good learning ability for this variety of forms of the bladder. The location of the pubic bone and abdomen still needed to be added to the images to enhance convolution learning.

**Figure 10:**
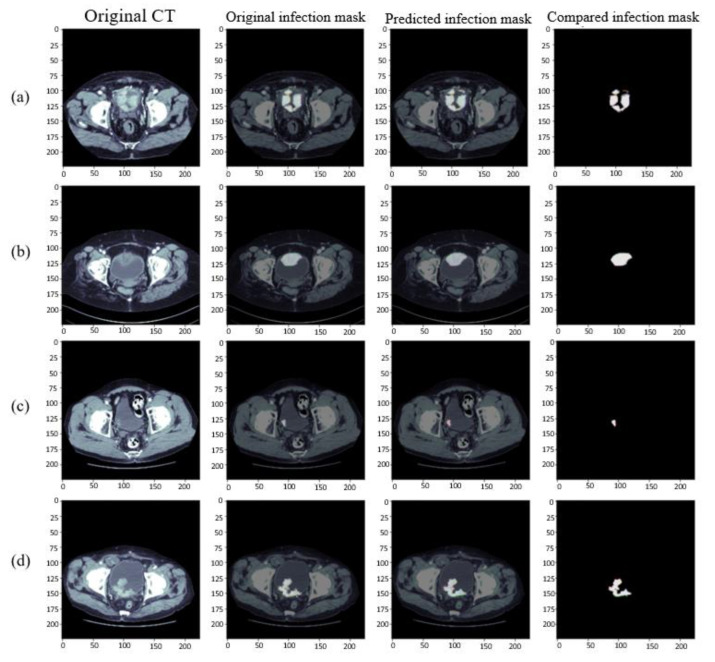
Results of segmentation of bladder lesions in our model. From left to right, Original CT images, Ground Truth, the prediction results, and the superposition comparison of Ground Truth and prediction results. (**a**) Multiple large tumors, (**b**) large tumors, (**c**) small tumors, and (**d**) irregular tumors.

Cystic lesions had the same diverse morphology as bladder lesions. As shown in Figure 10, the most common lesions were divided into the following four types and their combinations. Figure 10a shows a lesion formed by multiple large tumors. Its main prediction results were good, but there was no tumor location in the upper right corner of many large tumors. Our network prediction results mostly judge that there was a tumor here; in Figure 10b,c, the round lesions in the round bladder, irregular bladder, and small tumors in the bladder could be predicted excellently by using RDA U-Net convolution. Finally, the irregular tumor type in Figure 10d could be observed from the comparison chart of the last Ground Truth and the prediction results, indicating that this type of lesion could also be excellently predicted. However, due to the diversity of bladder organs and lesions, the training segmentation could be improved by increasing the amount of data to achieve better results.

**Figure 11:**
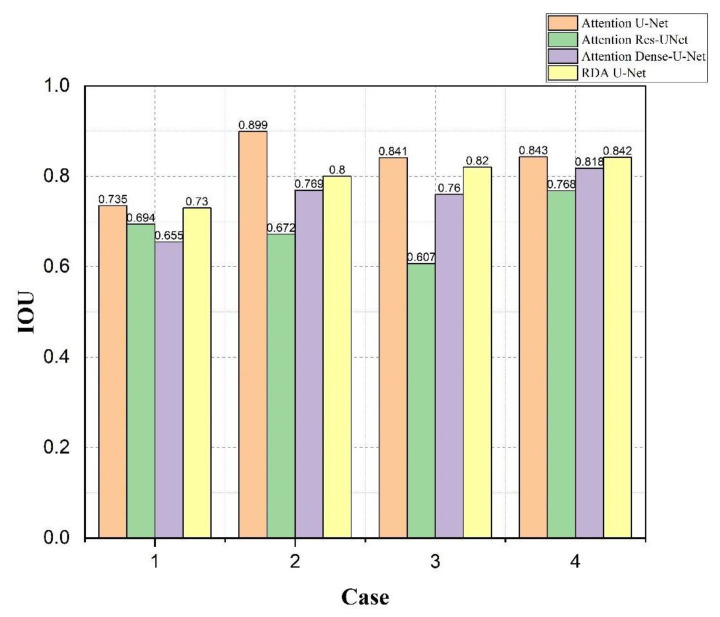
Using Attention U-Net, Attention Res U-Net, Attention Dense U-Net, and our RDA U-Net to compare the *IoU* values of bladder lesions in 4 cases.

**Figure 12:**
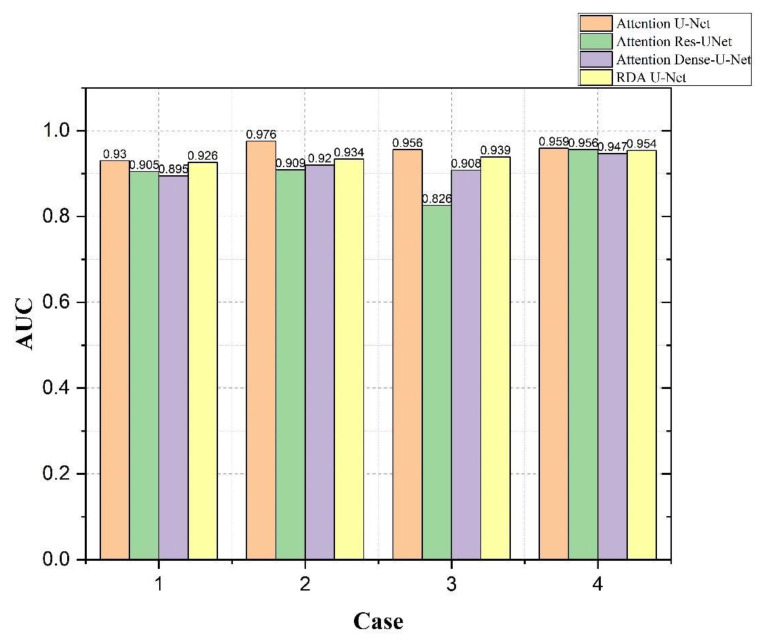
Using Attention U-Net, Attention Res U-Net, Attention Dense U-Net, and our RDA U-Net to compare AUC values of bladder lesions in 4 cases.

The results of bladder lesions in four different convolutions, Attention U-Net, Attention Res U-Net, Attention Dense U-Net, and our RDA U-Net, were compared in four other cases, as shown in Figure 11 and Figure 12. It was observed in Figure 11 that the results of our convolution in four cases were close to the best value. However, there was a significant difference between the values of Case 2 and Attention U-Net in small lesions and those of the same convolution in Case 2 and Case 3; our convolution was also much higher than Attention Res U-Net. The overall difference between the results of the area under the curve (AUC) [40] in Figure 12 and other evaluation indicators was smaller. In Case 3, a small lesion in an irregular bladder, our convolution was better than that of the other two. Although Case 4 was slightly lower than Attention Res-U-Net in a patchy lesion, all convolutions, in this case, have little difference, as explained in the following figure.

**Figure 13:**
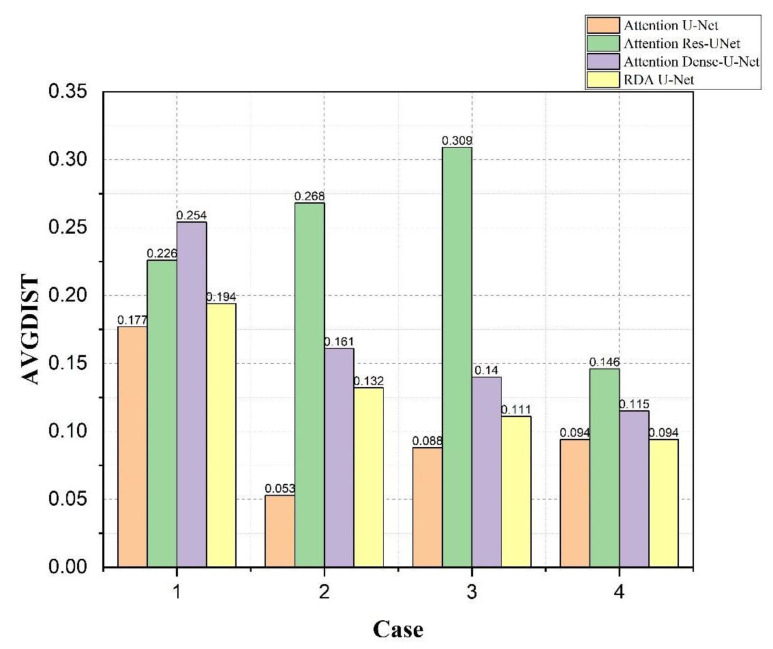
Using Attention U-Net, Attention Res U-Net, Attention Dense U-Net, and our RDA U-Net to compare the AVGDIST values of bladder lesions in 4 cases.

In Figure 13, the lower the *AVGDIST* value shown, the closer the lesion edge was, and the better the result value was. From Case 2, we knew that Attention U-Net had excellent training results for smaller lesions in the bladder. In this case, we also observed that our convolution was much better than Attention Res U-Net. The results of Attention Res U-Net in Case 3 showed that this convolution was not suitable for small lesions in the bladder. Finally, it was found in Case 4. However, our convolution was slightly lower than Attention Res U-Net in Figure 12; our convolution had the same *AVGDIST* as the Attention U-Net model with excellent bladder training. This result showed that our convolution had similar training ability in similar lesions.

From Figure 11, Figure 12 and Figure 13, it could be observed that our network was better than the other two convolutional networks. Most cases had similar results with the bladder training excellent Attention U-Net.

## 5. Conclusions

This paper proposed the RDA U-Net model architecture, which automatically segments organs and lesions in CT images. This model used ResBlock and DenseBlock at the encoder position so that the overall network had sufficient feature maps and parameters but did not require a long computing time. At the decoder position, Attention Gates were used to help the model suppress irrelevant areas of the image while focusing on useful salient features. The proposed model provided satisfactory bladder and lesion segmentation results. In the bladder image, we used 41 patients provided by TCGA and KMU for training. The ACC values of bladder and lesion were 0.9656 and 0.9394, respectively. The operation time was reduced by about 44% compared to other convolutions. IoU values were 0.9505 and 0.8024, respectively. *AVGDIST* was as low as 0.02 and 0.12, respectively. We used less time to complete the training and obtained approximate accuracy. Our convolution could achieve excellent results and take less time to complete the training.

1. By changing the HU value, we made the picture clearer without a contrast agent. Through experiments, we confirmed that the training speed of our model was much lower than that of other models.
2. Through experiments, it was confirmed that RDA U-NET reduced the computing time by about 40% compared with the proposed strategy.
3. With the data from Kaohsiung Medical University and Open-Source Imaging (TCGA), the accuracy of RDA U-Net in bladder organ identification and lesion identification reached 96% and 93%, respectively.

The advantage of RDA U-Net is that it can clearly distinguish CT images without a contrast agent, eliminating the side effects of contrast agents and making more people accept this technology. Another advantage is that it is fast and accurate, which is a great advantage for the clinical treatment of cancer. However, meeting the requirements of RDA U-Net requires relatively expensive computer equipment and many training materials to maintain a good level of training results.

In the future, it can be used for different medical image formats such as MRI, ultrasound, etc. With the improvement of computer hardware, three-dimensional (3D) CNN may gain more significant advantages than two-dimensional (2D) CNN. The 2D CNN model algorithm is a crucial way to achieve future goals once the 3D CNN network can help doctors obtain more information on organs and lesions.

## Figures and Tables

**Table 1:** Comparison of bladder model parameters and training time.

Method	Parameter	Bladder Tumor-Training Time (s)	Bladder-Training Time (s)
RDA U-Net	13,053,861	41	42
Attention U-Net [32]	35,238,293	56	57
Attention Dense-U-Net [38]	14,374,021	59	60
Attention Res-U Net [39]	12,981,573	40	40

**Table 2:** Results of bladder segmentation evaluated by different models.

Method	*ACC*	*DSC*	*IoU*	*AVGDIST*
RDA U-Net	0.9656	0.9745	0.9505	0.0269
Attention U-Net [32]	0.9610	0.9771	0.9553	0.0245
Attention Dense-U-Net [38]	0.9721	0.9811	0.9631	0.0187
Attention Res-U-Net [39]	0.9614	0.9717	0.9452	0.0291

*ACC*: Accuracy, *DSC*: Dice Score Coefficient, *IoU*: Intersection over Union, and *AVGDIST*: Average Hausdorff Distance.

**Table 3:** Results of bladder lesions segmentation evaluated by different models.

Method	*ACC*	*DSC*	*IoU*	*AVGDIST*
RDA U-Net	0.9394	0.8895	0.8024	0.1279
Attention U-Net [32]	0.9330	0.8993	0.8184	0.1141
Attention Dense-U-Net [38]	0.9258	0.8797	0.7869	0.1406
Attention Res-U-Net [39]	0.8928	0.8322	0.7150	0.2080

*ACC*: Accuracy, *DSC*: Dice Score Coefficient, *IoU*: Intersection over Union, and *AVGDIST*: Average Hausdorff Distance.

## Data Availability

The data presented in this study are available on request from the corresponding author.
